# A case of constrictive pericarditis with aortic insufficiency: the role of cardiac magnetic resonance imaging

**DOI:** 10.1007/s10554-025-03373-2

**Published:** 2025-03-10

**Authors:** Juthipong Benjanuwattra, Nicholas Biondi, Jonathan D. Feazell, Sara A. Dressman, David M. Harris

**Affiliations:** 1 Division of Cardiology, University of Cincinnati, Cincinnati, OH USA; 2 Division of Cardiology, University of Kentucky, Lexington, KY USA; 3 Division of Internal Medicine, University of Cincinnati, Cincinnati, OH USA

**Keywords:** Aortic regurgitation, Constrictive pericarditis, Cardiac magnetic resonance imaging, Echocardiography, Imaging

## Abstract

Transthoracic echocardiography is recommended as a diagnostic test of choice for constrictive pericarditis. Nevertheless, several limitations exist. Hereby, we present the role of cardiac magnetic resonance imaging in a case of constrictive pericarditis with concurrent aortic insufficiency that masked the echocardiographic features of ventricular interdependence.

## Case presentation

A 50-year-old female patient with relapsed Hodgkin’s lymphoma initially presented with bilateral pleural effusions and a large pericardial effusion necessitating pericardiocentesis and subsequent pericardial window. She underwent allogenic bone marrow transplant which was complicated by neutropenic fever and a prolonged hospitalization. A month later, she developed gradually worsening dyspnea, positional chest pain and recurrent bilateral pleural effusions requiring multiple thoracenteses. Given her history and symptomatology, there was concern for constrictive physiology.

**Fig. 1:**
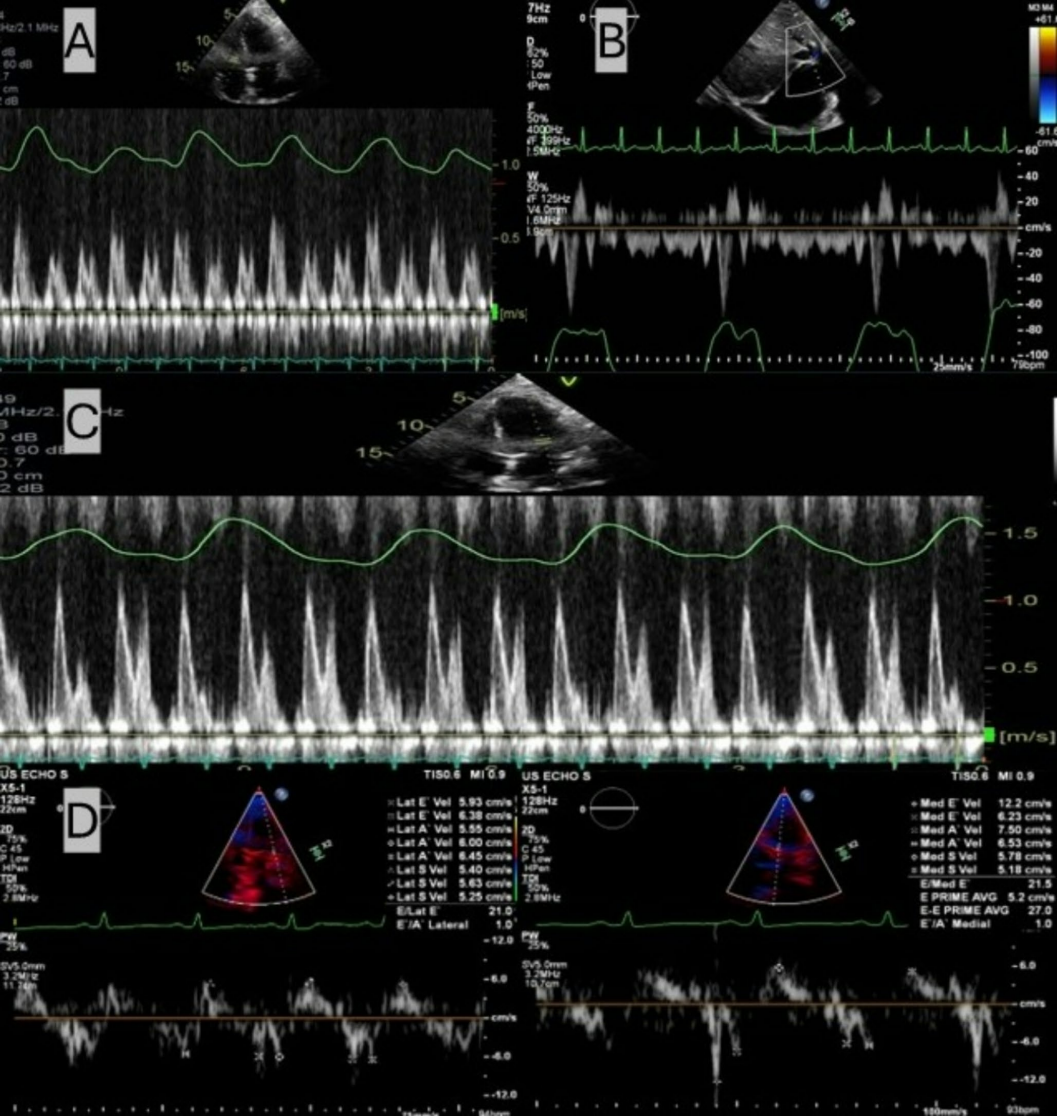
Transthoracic pulsed-wave Doppler analysis. (**A**) apical 4-chamber view showing respirophasic change of the tricuspid inflow velocities; (**B**) subcostal view showing pleural effusion and hepatic venous Doppler with expiratory diastolic flow reversal and increased forward flow; (**C**) apical 4-chamber view showing the mitral inflow velocities without respirophasic change; (**D**) tissue Doppler showing annulus reversus

**Fig. 2:**
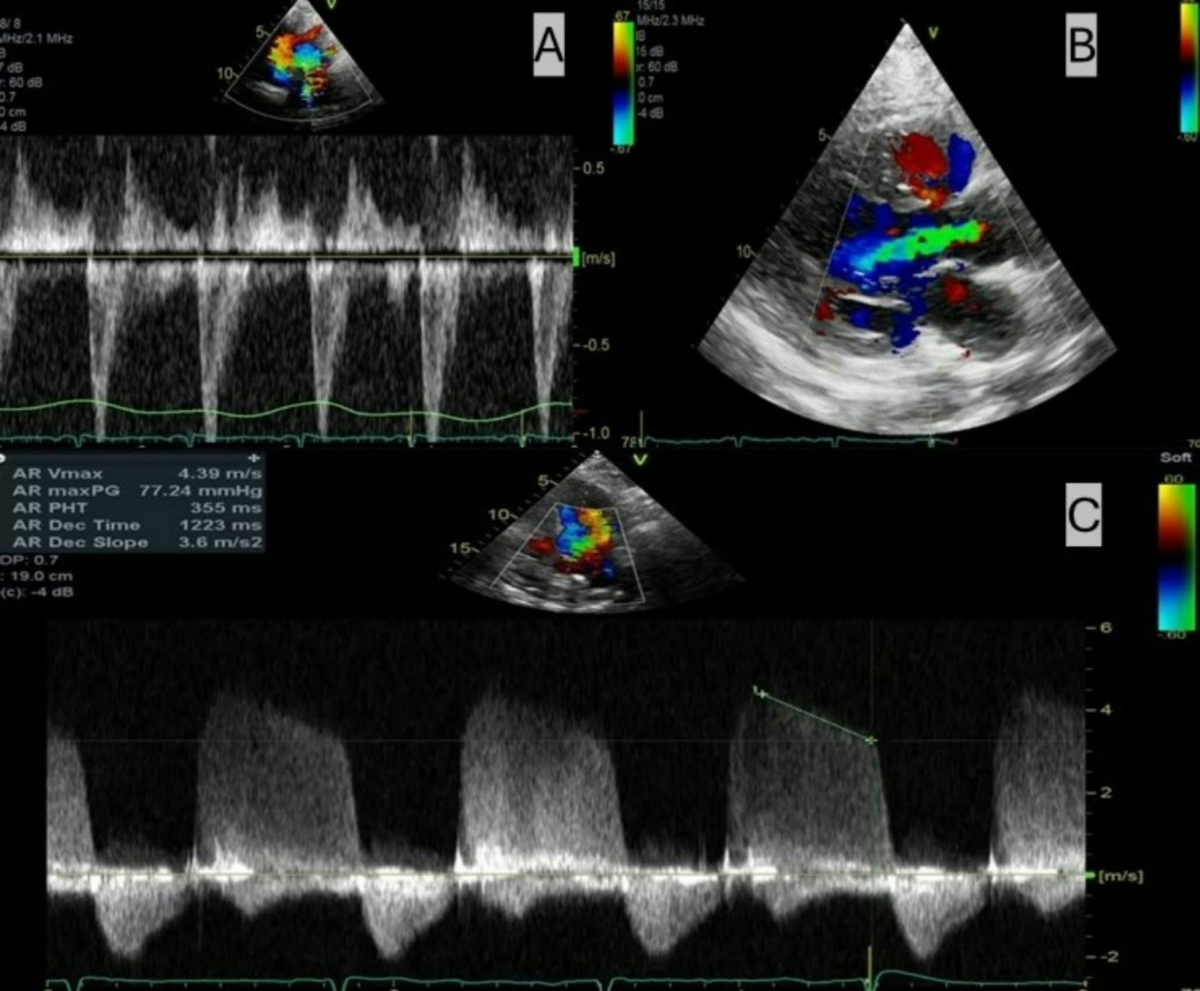
Transthoracic Doppler analysis. (**A**) suprasternal view showing holodiastolic flow reversal of the descending aorta; (**B**) parasternal long-axis showing vena contracta of 5.5 mm; (**C**) apical 5-chamber view showing aortic regurgitation with pressure half-time of 355 msec

The transthoracic echocardiogram (TTE) showed significant respirophasic variation in the tricuspid inflow velocities (Fig. 1A), the hepatic venous Doppler with expiratory diastolic flow reversal and increased forward flow as well as a dilated vena cava and pleural effusions (Fig. 1B). However, there was no variation in the mitral inflow velocities (Fig. 1C). Annulus reversus was demonstrated on tissue Doppler (Fig. 1D). Respirophasic septal shift was subtle without clear interdependence. There was also moderate-severe aortic regurgitation (AR) with a vena contracta measuring 5.5 mm, pressure half-time of 355 msec, and holodiastolic flow reversal in the descending aorta (Fig. 2A-C). Left ventricular volumetrics were within normal limits.

**Fig. 3:**
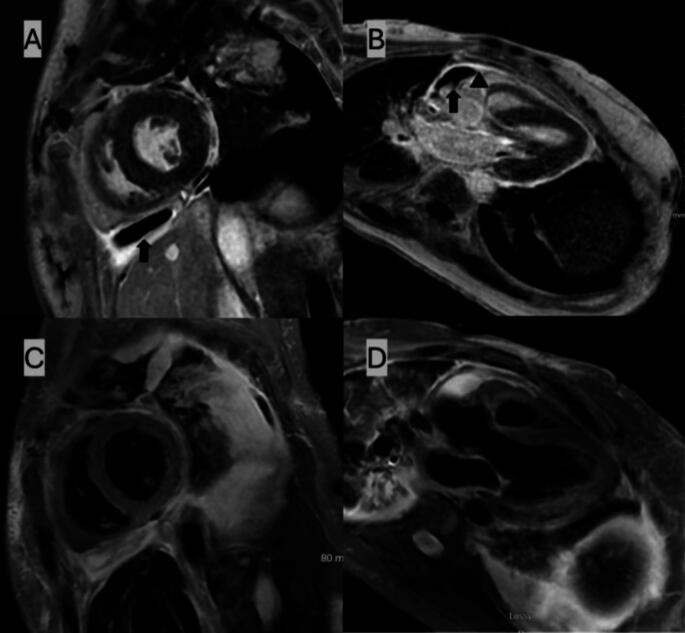
A diagnosis of effusive constrictive pericarditis was made via CMR. (**A**) short axis image showing thickened pericardium measuring 3.2 mm with gadolinium enhancement (arrowhead) and pericardial effusion (arrow); (**B**) long axis image showing thickened pericardium (arrowhead) with loculated pericardial effusion (arrow) and aortic regurgitation; (**C**, **D**) T2-weighted dark blood images showing hyperintense pericardial effusion without myocardial edema

**Fig. 4:**
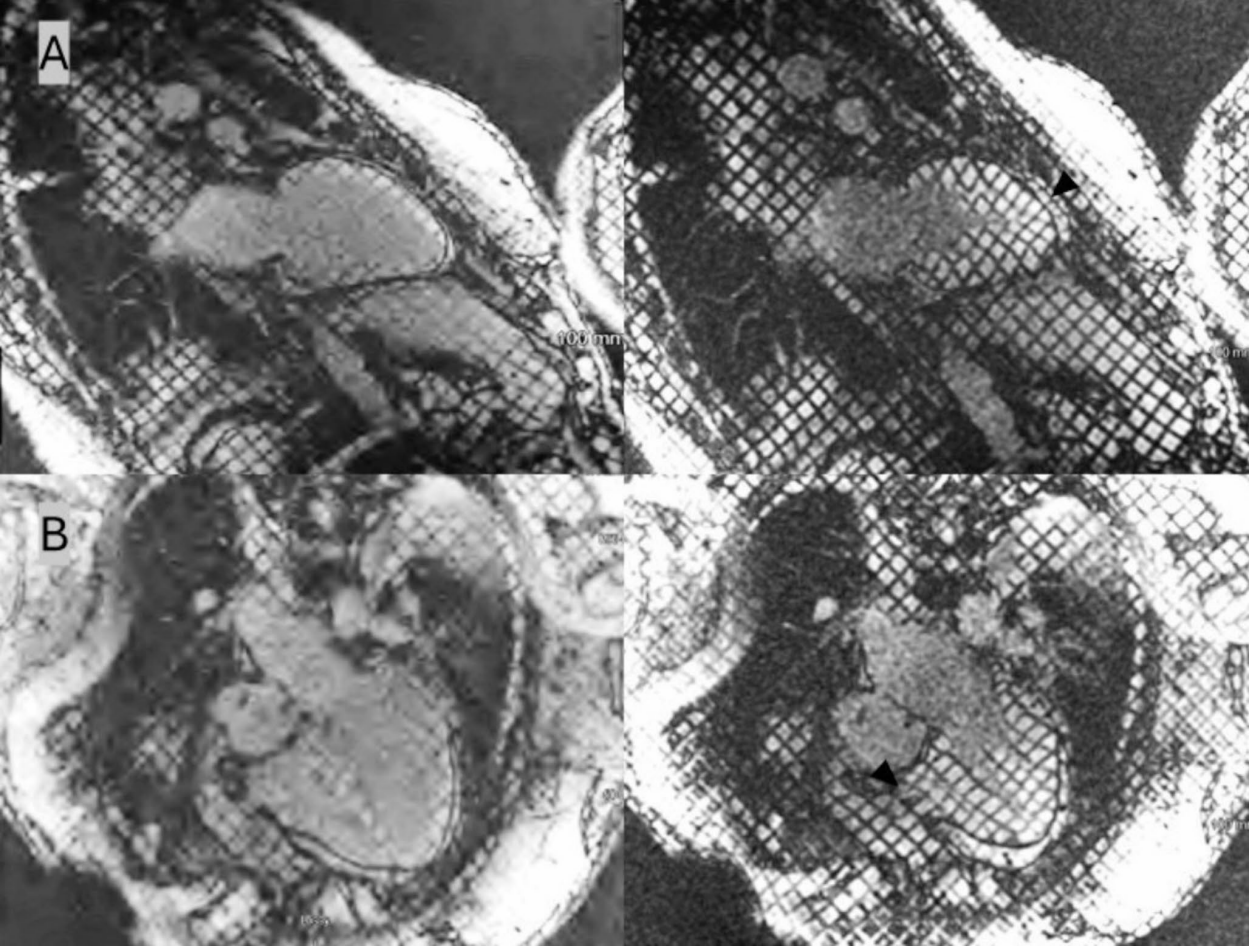
Tagged images showing tethering of the anterior left ventricular (**A**) and right ventricular wall (**B**) as evidenced by the continuity of tagged signals during systole (arrowhead)

Due to discrepant TTE findings and a high clinical suspicion, cardiac magnetic resonance imaging (CMR) was obtained. The patient was intubated and ventilated for the study due to significant shortness of breath and anxiety. As illustrated in Fig. 3, there was diffuse non-calcified pericardial thickening measuring 3.2 mm with extensive late gadolinium enhancement (LGE). Spatial modulation of magnetization (SPAMM) was used for tagged imaging and revealed evidence of myocardial adhesions (Fig. 4). There was no evidence of diastolic septal bounce or respirophasic septal shift. The aortic regurgitant fraction was 26%. An invasive hemodynamic study was not performed due to the patient’s preference.

## Follow-up

The patient was eventually diagnosed with effusive-constrictive pericarditis. She was started on a course of colchicine and prednisone. Prednisone, rather than high dose aspirin, was used given the concurrent oncological indication. Colchicine was discontinued after one month due to significant gastrointestinal side-effects. Prednisone was slowly tapered off over 10 weeks. On follow up, the patient was free of dyspnea and chest pain.

## Discussion

Constrictive pericarditis (CP) is an uncommon entity predominantly manifesting as right-sided heart failure resulting from a fixed, thickened, fibrotic pericardium and subsequently impaired ventricular filling [1]. Most cases are idiopathic or iatrogenic following cardiac surgery; however, tuberculosis remains the leading cause in developing countries. Other etiologies include connective tissue disease, radiation, malignancy, or uremia [1, 2].

The pericardium, even healthy and non-diseased, contributes substantially to diastolic ventricular interdependence, a phenomenon in which the filling of one ventricle affects the compliance and geometry of the other ventricle [3]. An exaggerated inspiratory fall of systolic blood pressure of greater than 10 mmHg, or pulsus paradoxus, can be seen in various conditions including CP, asthmatic exacerbation, right ventricular infarction, and pulmonary embolism [4, 5]. A dissociation of the intrathoracic and intracardiac pressures due to pericardial constraint leads to pooling of blood in the pulmonary vasculature and loss of pressure gradient with subsequent underfilling of the left ventricle (LV) during inspiration. LV underfilling is exacerbated by the filling of right ventricle (RV) and a resultant leftward septal shift [4].

TTE is usually an initial imaging study of choice for a diagnosis of CP, although not without limitation. Pericardial thickness is often not easily measured by TTE [6]. Image quality can also be limited by body habitus, lung disease, and intervening air space after cardiac surgery. Despite optimal image quality, TTE results from our patient were inconclusive. Evidence of ventricular interdependence, respirophasic variation in septal shift and mitral inflow velocities, was absent in our patient. The lack of interdependence may be explained by hemodynamic changes associated with AR with resultant elevated left ventricular filling pressures and dampening of the effect from respiratory variation [5]. Though, it is possible that the constrictive process may not be uniform, thus classic hemodynamic hallmarks may not always be present.

Owing to its high spatial and temporal resolution, cine CMR provides excellent anatomic detail and tissue characterization, in addition to functional evaluation of the hemodynamics associated with CP [7]. It has proven to be an excellent non-invasive modality for the diagnosis CP given its excellent sensitivity and specificity. To rule out right ventricular failure, a condition sharing similar clinical presentation with CP, the use of cine CMR with steady-state free precession (SSFP) is the gold standard to quantify cardiac volume and ejection fraction of both ventricles [7].

In a retrospective cohort study of patients with surgically-proven CP, a model incorporating both relative interventricular septal excursion and pericardial thickness (≥ 3 mm) exhibited 100% sensitivity and 90% specificity in diagnosing CP [8]. Other parameters include diastolic septal bounce and eccentricity index, both of which are a manifestation of ventricular interdependence [8].

Tagged cine CMR, which utilizes SPAMM, is a useful tool to evaluate fibrotic adhesion of pericardial layers [9]. In normal pericardium, the shear motion between pericardial layers leads to the displacement of tag continuity [7]. The persistent continuity of tagged signals between the pericardium and myocardium throughout cardiac cycles signifies an adhesion to the myocardium as seen in our patient [9].

The presence and extent of pericardial inflammation and fibrosis can be readily assessed with the use of gadolinium [1]. It was shown by a small pilot study that the presence of LGE was associated with reversibility and positive response to anti-inflammatory therapy [10]. The presence of myocardial LGE, which can be seen in myopericarditis or restrictive cardiomyopathy (RCM) is associated with worse outcomes. It is of paramount importance to differentiate CP from RCM as the treatment strategies are vastly different [7]. Although biatrial enlargement is a common finding in both CP and RCM, the relative atrial volume ratio, as defined by the left atrial volume divided by right atrial volume, may help differentiate CP from RCM with sensitivity of 82.6% and specificity of 86.4% using a cut-off value of > 1.32 [11].

CMR was integral in confirming the diagnosis of CP for this patient whose echocardiographic findings were inconclusive. It clearly characterized a thickened pericardium with LGE and evidence of tethering on tagged images. Additionally, the severity of AR was also quantified, and a loculated pericardial effusion was identified. The location and extent of pericardial fluid is easily assessed by CMR. It was previously suggested that effusive-constrictive pericarditis is likely an evolution of acute pericarditis progressing to eventual chronic constrictive pericarditis without effusion [12].

## Conclusion

Constrictive pericarditis is an uncommon condition associated with significant morbidity and mortality that is often overlooked due to its diagnostic difficulty. The role of multimodality imaging including CMR should be emphasized to allow a prompt diagnosis and treatment.

## Data Availability

No datasets were generated or analysed during the current study.
